# Supplementary material for: Deletion of wfs1 Impairs Oligodendrocyte Precursor Cells Dorsal Distribution and Myelination Through the wfs1-hmgcs1 Axis in Zebrafish
Source: Biology (Basel). 2026 Aug 16;15(16):1402. doi: 10.3390/biology15161402 (PMC13510151; doi:10.3390/biology15161402)
Supplement: Supplementary file 1 [file biology-15-01402-s001.zip › File S1-Transcriptome sequencing data and differentially expressed genes (DEGs) in wfs1-- zebrafish at 5 dpf/2.KEGG/wfs1KOvsWT/GSEA/wfs1KO/PPAR_SIGNALING_PATHWAY/PPAR_SIGNALING_PATHWAY(DRE03320).html]

Details for gene set PPAR\_SIGNALING\_PATHWAY(DRE03320)[GSEA]

|  || Dataset | deg.sample |
| Phenotype | sample.cls |
| Upregulated in class | wfs1KO |
| GeneSet | PPAR\_SIGNALING\_PATHWAY(DRE03320) |
| Enrichment Score (ES) | 0.25689915 |
| Normalized Enrichment Score (NES) | 1.30873 |
| Nominal p-value | 0.084848486 |
| FDR q-value | 0.2827995 |
| FWER p-Value | 0.957 |
Table: GSEA Results Summary

  

Fig 1: Enrichment plot: PPAR\_SIGNALING\_PATHWAY(DRE03320)      
 Profile of the Running ES Score & Positions of GeneSet Members on the Rank Ordered List

  

| PROBE | DESCRIPTION (from dataset) | GENE SYMBOL | GENE\_TITLE | RANK IN GENE LIST | RANK METRIC SCORE | RUNNING ES | CORE ENRICHMENT || 1 | 334529 | aqp7 |  |  | 389 | 3.323 | 0.0300 | Yes |
| 2 | 406473 | pck2 |  |  | 487 | 3.031 | 0.0654 | Yes |
| 3 | 30355\_1 | apoa1a |  |  | 615 | 2.745 | 0.0963 | Yes |
| 4 | 550134\_1 | ubb |  |  | 694 | 2.603 | 0.1269 | Yes |
| 5 | novel.905 | - |  |  | 864 | 2.303 | 0.1509 | Yes |
| 6 | 553512 | slc27a1b |  |  | 1051 | 2.047 | 0.1710 | Yes |
| 7 | 140615 | fads2 |  |  | 1590 | 1.600 | 0.1744 | Yes |
| 8 | 550432 | slc27a4 |  |  | 1646 | 1.569 | 0.1926 | Yes |
| 9 | 100005717 | cpt2 |  |  | 1666 | 1.560 | 0.2118 | Yes |
| 10 | 777766 | ubc |  |  | 1935 | 1.395 | 0.2211 | Yes |
| 11 | 436636 | cd36 |  |  | 2057 | 1.345 | 0.2344 | Yes |
| 12 | 394156\_1 | acadl |  |  | 2548 | 1.160 | 0.2337 | Yes |
| 13 | 393622 | acsl4a |  |  | 3304 | 0.972 | 0.2223 | Yes |
| 14 | 568707 | afp4 |  |  | 3364 | 0.961 | 0.2327 | Yes |
| 15 | 558239 | si:ch211-113j14.1 |  |  | 3492 | 0.932 | 0.2405 | Yes |
| 16 | 393543 | ilk |  |  | 3651 | 0.902 | 0.2470 | Yes |
| 17 | 171478 | fabp3 |  |  | 3698 | 0.893 | 0.2569 | Yes |
| 18 | 100000859 | ehhadh |  |  | 4565 | 0.737 | 0.2390 | No |
| 19 | 562602 | adipoqa |  |  | 4938 | 0.689 | 0.2361 | No |
| 20 | 100006023 | acsbg1 |  |  | 5003 | 0.680 | 0.2427 | No |
| 21 | 394148 | cyp7a1 |  |  | 5651 | 0.622 | 0.2302 | No |
| 22 | 447860 | acsl5 |  |  | 5835 | 0.604 | 0.2321 | No |
| 23 | 751715 | adipoqb |  |  | 6009 | 0.585 | 0.2341 | No |
| 24 | 445281 | cyp8b1 |  |  | 6109 | 0.576 | 0.2383 | No |
| 25 | 541410 | slc27a1a |  |  | 6267 | 0.560 | 0.2405 | No |
| 26 | 449662 | acox1 |  |  | 6280 | 0.559 | 0.2472 | No |
| 27 | 30708 | fabp2 |  |  | 6389 | 0.548 | 0.2508 | No |
| 28 | 794841\_2 | plin2 |  |  | 7122 | 0.480 | 0.2339 | No |
| 29 | 794841 | plin2 |  |  | 7215 | 0.472 | 0.2370 | No |
| 30 | 553734 | scdb |  |  | 7381 | 0.461 | 0.2376 | No |
| 31 | 555578 | rxraa |  |  | 8324 | 0.411 | 0.2132 | No |
| 32 | 768202 | pdpk1a |  |  | 9086 | 0.361 | 0.1939 | No |
| 33 | 553579 | fabp11b |  |  | 9120 | 0.359 | 0.1974 | No |
| 34 | 101884800 | LOC101884800 |  |  | 9188 | 0.353 | 0.1998 | No |
| 35 | 436617 | rxrgb |  |  | 9377 | 0.342 | 0.1982 | No |
| 36 | 548341 | nr1h3 |  |  | 9419 | 0.339 | 0.2012 | No |
| 37 | 431754 | acaa1 |  |  | 9613 | 0.326 | 0.1993 | No |
| 38 | 322341 | cyp27a1.4 |  |  | 10296 | 0.285 | 0.1815 | No |
| 39 | 560000 | cpt1ab |  |  | 10472 | 0.274 | 0.1794 | No |
| 40 | 768196 | me1 |  |  | 10781 | 0.256 | 0.1730 | No |
| 41 | 394060 | hmgcs1 |  |  | 11015 | 0.243 | 0.1688 | No |
| 42 | 559097 | cyp7a1b |  |  | 11302 | 0.227 | 0.1626 | No |
| 43 | 559656 | acsl3b |  |  | 11481 | 0.218 | 0.1598 | No |
| 44 | 449677 | cpt1b |  |  | 11572 | 0.212 | 0.1597 | No |
| 45 | 415166 | fabp6 |  |  | 11715 | 0.205 | 0.1578 | No |
| 46 | 445125 | pltp |  |  | 11733 | 0.204 | 0.1599 | No |
| 47 | 565876 | si:dkey-91i10.3 |  |  | 11920 | 0.195 | 0.1565 | No |
| 48 | 406421 | acox3 |  |  | 12372 | 0.170 | 0.1445 | No |
| 49 | 407736 | fabp7b |  |  | 12467 | 0.166 | 0.1436 | No |
| 50 | 566863 | acsl6 |  |  | 12701 | 0.154 | 0.1382 | No |
| 51 | 100101640 | apoa1b |  |  | 13476 | 0.117 | 0.1154 | No |
| 52 | 550361 | cpt1cb |  |  | 13535 | 0.114 | 0.1150 | No |
| 53 | 494096 | zgc:101540 |  |  | 14569 | 0.066 | 0.0833 | No |
| 54 | 447944 | fabp11a |  |  | 15100 | 0.041 | 0.0672 | No |
| 55 | 554095 | fabp1b.1 |  |  | 15359 | 0.030 | 0.0594 | No |
| 56 | 394156 | acadl |  |  | 15452 | 0.024 | 0.0569 | No |
| 57 | 568835 | slc27a6 |  |  | 15475 | 0.023 | 0.0565 | No |
| 58 | 30754 | ppardb |  |  | 16357 | -0.013 | 0.0289 | No |
| 59 | 334561 | acsl4b |  |  | 16648 | -0.022 | 0.0201 | No |
| 60 | 445175 | acsl1b |  |  | 16699 | -0.023 | 0.0188 | No |
| 61 | 100004228 | slc27a2b |  |  | 17093 | -0.036 | 0.0069 | No |
| 62 | 558088 | cpt1aa |  |  | 17283 | -0.044 | 0.0015 | No |
| 63 | 568883 | LOC568883 |  |  | 17502 | -0.054 | -0.0047 | No |
| 64 | 555308 | acsl1a |  |  | 18027 | -0.077 | -0.0202 | No |
| 65 | 30354 | lpl |  |  | 18171 | -0.084 | -0.0236 | No |
| 66 | 378727 | pck1 |  |  | 19075 | -0.125 | -0.0504 | No |
| 67 | 100136866 | zgc:172295 |  |  | 19211 | -0.131 | -0.0530 | No |
| 68 | 567157 | acsl3a |  |  | 19451 | -0.142 | -0.0587 | No |
| 69 | 335159 | acsbg2 |  |  | 19541 | -0.145 | -0.0597 | No |
| 70 | 393831 | dbi |  |  | 19840 | -0.159 | -0.0671 | No |
| 71 | 100004274 | cyp8b2 |  |  | 21320 | -0.232 | -0.1106 | No |
| 72 | 100332846 | acsl2 |  |  | 21878 | -0.261 | -0.1249 | No |
| 73 | 492647 | angptl4 |  |  | 22123 | -0.274 | -0.1290 | No |
| 74 | 557714 | pparab |  |  | 22357 | -0.287 | -0.1327 | No |
| 75 | 557037 | pparg |  |  | 22764 | -0.311 | -0.1415 | No |
| 76 | 100002756 | sorbs1 |  |  | 22986 | -0.326 | -0.1444 | No |
| 77 | 492719 | me3 |  |  | 23069 | -0.332 | -0.1427 | No |
| 78 | 58128 | fabp7a |  |  | 23131 | -0.336 | -0.1404 | No |
| 79 | 406283 | acadm |  |  | 23289 | -0.347 | -0.1409 | No |
| 80 | 30750 | pparda |  |  | 24113 | -0.400 | -0.1617 | No |
| 81 | 555308\_1 | acsl1a |  |  | 24418 | -0.416 | -0.1660 | No |
| 82 | 791610 | fabp1a |  |  | 24549 | -0.420 | -0.1647 | No |
| 83 | 563298 | pparaa |  |  | 24597 | -0.421 | -0.1609 | No |
| 84 | 100333227 | LOC100333227 |  |  | 25306 | -0.467 | -0.1772 | No |
| 85 | 568656 | LOC568656 |  |  | 25931 | -0.524 | -0.1902 | No |
| 86 | 403000 | pdpk1b |  |  | 26060 | -0.534 | -0.1874 | No |
| 87 | 447848 | cyp8b3 |  |  | 26136 | -0.541 | -0.1829 | No |
| 88 | 393839 | scp2a |  |  | 26251 | -0.552 | -0.1795 | No |
| 89 | 30708\_1 | fabp2 |  |  | 26433 | -0.570 | -0.1779 | No |
| 90 | 386661 | scd |  |  | 26590 | -0.587 | -0.1754 | No |
| 91 | 30464 | rxrga |  |  | 26695 | -0.598 | -0.1711 | No |
| 92 | 30530 | rxrba |  |  | 27585 | -0.683 | -0.1904 | No |
| 93 | 550432\_1 | slc27a4 |  |  | 28306 | -0.788 | -0.2030 | No |
| 94 | 449925 | slc27a2a |  |  | 29093 | -0.940 | -0.2158 | No |
| 95 | 100331214 | LOC100331214 |  |  | 29706 | -1.130 | -0.2207 | No |
| 96 | 406473\_1 | pck2 |  |  | 29767 | -1.153 | -0.2079 | No |
| 97 | 541410\_1 | slc27a1a |  |  | 29803 | -1.165 | -0.1942 | No |
| 98 | 402831 | cyp27a7 |  |  | 30086 | -1.279 | -0.1869 | No |
| 99 | 560000\_1 | cpt1ab |  |  | 30321 | -1.399 | -0.1764 | No |
| 100 | 100009650 | zgc:158482 |  |  | 30362 | -1.423 | -0.1596 | No |
| 101 | 795106 | cyp27a1.2 |  |  | 30481 | -1.495 | -0.1443 | No |
| 102 | 100005717\_1 | cpt2 |  |  | 30500 | -1.505 | -0.1258 | No |
| 103 | 30355 | apoa1a |  |  | 31222 | -2.267 | -0.1197 | No |
| 104 | 794841\_1 | plin2 |  |  | 31571 | -3.132 | -0.0908 | No |
| 105 | 723999 | zgc:136333 |  |  | 31649 | -3.456 | -0.0493 | No |
| 106 | 550134 | ubb |  |  | 31783 | -4.479 | 0.0034 | No |
Table: GSEA details [plain text format]

  

Fig 2: PPAR\_SIGNALING\_PATHWAY(DRE03320)      
 Blue-Pink O' Gram in the Space of the Analyzed GeneSet

  

Fig 3: PPAR\_SIGNALING\_PATHWAY(DRE03320): Random ES distribution      
 Gene set null distribution of ES for **PPAR\_SIGNALING\_PATHWAY(DRE03320)**
